# Antibiotic-Resistant Bacteria in Aquaculture and Climate Change: A Challenge for Health in the Mediterranean Area

**DOI:** 10.3390/ijerph18115723

**Published:** 2021-05-26

**Authors:** Milva Pepi, Silvano Focardi

**Affiliations:** 1 Stazione Zoologica Anton Dohrn, Fano Marine Centre, Viale Adriatico 1-N, 61032 Fano, Italy; milva.pepi@szn.it; 2 Department of Environmental Sciences, Università di Siena, Via Mattioli, 4, 53100 Siena, Italy

**Keywords:** aquaculture, antibiotic-resistance, climate change, Mediterranean Sea, One Health

## Abstract

Aquaculture is the productive activity that will play a crucial role in the challenges of the millennium, such as the need for proteins that support humans and the respect for the environment. Aquaculture is an important economic activity in the Mediterranean basin. A great impact is presented, however, by aquaculture practices as they involve the use of antibiotics for treatment and prophylaxis. As a consequence of the use of antibiotics in aquaculture, antibiotic resistance is induced in the surrounding bacteria in the column water, sediment, and fish-associated bacterial strains. Through horizontal gene transfer, bacteria can diffuse antibiotic-resistance genes and mobile resistance genes further spreading genetic determinants. Once triggered, antibiotic resistance easily spreads among aquatic microbial communities and, from there, can reach human pathogenic bacteria, making vain the use of antibiotics for human health. Climate change claims a significant role in this context, as rising temperatures can affect cell physiology in bacteria in the same way as antibiotics, causing antibiotic resistance to begin with. The Mediterranean Sea represents a ‘hot spot’ in terms of climate change and aspects of antibiotic resistance in aquaculture in this area can be significantly amplified, thus increasing threats to human health. Practices must be adopted to counteract negative impacts on human health, with a reduction in the use of antibiotics as a pivotal point. In the meantime, it is necessary to act against climate change by reducing anthropogenic impacts, for example by reducing CO_2_ emissions into the atmosphere. The One Health type approach, which involves the intervention of different skills, such as veterinary, ecology, and medicine in compliance with the principles of sustainability, is necessary and strongly recommended to face these important challenges for human and animal health, and for environmental safety in the Mediterranean area.

## 1. Introduction

Aquaculture is the productive industrial activity that will play a crucial role in providing solutions to the millennium challenges [1]. Fish and seafood consumption will increase by 27%, according to expectations for aquaculture for 2030, in which a doubling of fish production is expected [2]. Aquaculture is a strategic sector for the years ahead, providing food required for a rapidly growing human population, taking into account the need to reduce impacts for health and for the environment during food production. These goals are designed based on the 2030 UN Agenda of Sustainable Development Goals (SDGs), which aims to provide people with food and protect the Planet from degradation [3]. The definition of aquaculture given by the FAO (Food and Agriculture Organization of the United Nations) is reported as follows: “*aquaculture is the farming of aquatic organisms including fish, mollusks, crustaceans, and aquatic plants*”. Farming implies some sort of intervention in the rearing process to enhance production, such as regular stocking, feeding, and protection from predators [4].

The Mediterranean Sea probably hosts the oldest coastal aquaculture system in the world, the Egyptian pond systems with a history of two to three millennia. Aquaculture has been known in Egypt for millennia, as evidenced by some friezes reported on tombs dating back to 2500 B.C., where the collection of tilapia from ponds was shown [5]. Shellfish culture in the Mediterranean area originated in France about 600 years ago [6]. Contemporary aquaculture in the Mediterranean area, began in the 1980s mostly with the cultivation of finfish, as the European sea bass (*Dicentrarchus labrax*) and gilthead sea bream (*Sparus aurata*), and shellfish. The production of shellfish in the Mediterranean is mainly represented by the European mussel (*Mytilus galloprovincialis*), the Japanese shell (*Ruditapes philippinarum*), the European flat oyster (*Ostrea edulis*), and the Pacific oyster (*Crassostrea gigas*). During the 1990s, the breeding of Atlantic bluefin tuna (*Thunnus thynnus*) was introduced, albeit with a limited expansion [7]. The global trend of Mediterranean aquaculture has evidenced a rapid growth over the years [8,9]). The continued increase in Mediterranean finfish aquaculture production was changed from small terrestrial sites to large enterprises along the coastline and to offshore sites [9]. In the Mediterranean area, almost all south European and north African countries carry out aquaculture activities, with the main productions in France, Greece, Italy, Spain, and Turkey. Legislation concerning aquaculture varies due to individual socio-economic and cultural differences among Mediterranean European and north African countries. Impacts on humans and the environmental health of aquaculture practices can be different among these regions [9,10]. In the Mediterranean and the Black Sea, in 2002, the total production was 133,936 tons of marine fish, whereas, in 2016, it increased to 307,171 tons of marine fish [11]. Greece is one of the major producers of finfish products in Europe, principally coming from near-shore and offshore marine aquaculture farms. In 2016, Greece produced 97,900 tons of marine finfish, mainly gilthead seabream and European seabass [11].

The use of antibiotics in aquaculture is well known and this practice can cause the spread of antibiotic residues in the marine environment, increasing the rates of antibiotic resistance in aquatic bacteria and, critically, transfer that resistance to human pathogens. In this review, a description of these important problems in the Mediterranean area is reported. It is also known that the onset of antibiotic resistance is due to high temperatures and this can further damage the Mediterranean area, where warming due to climate change is a growing concern. The One Health intervention as a possible way to counter this health challenge in the Mediterranean is proposed.

## 2. Antibiotic Use in Aquaculture

Antibiotics inhibit bacterial growth or destroy bacterial cells, representing essential drugs for human health since their discovery [12]. The European Medicines Agency (EMA) defined antibiotics as “… *any substance with a direct action on bacteria that is used for treatment or prevention of infections or infectious disease*” [13]. From the chemical point of view, antibiotics are complex molecules containing different functional groups within their formulae and are divided into different classes, according to their mechanisms of action. Antibiotics can act against bacteria via different mechanisms, including inhibition of cell wall synthesis, alteration of cell membranes, inhibition of protein synthesis, inhibition of nucleic acids synthesis, competitive antagonism, and antimetabolite activity [14]. In addition to their use in human medicine, antibiotics are widely used in veterinary medicine to improve animal health, with aquaculture being one of the most representative sectors. Antibiotics are used in aquaculture for the following purposes: (i) Prophylactic: The administration of medication to all animals in the lot to prevent diseases before they occur, with antibiotics used at sub-therapeutic exposure concentrations; (ii) Therapeutic: The administration of medication to treat sick animals; (iii) Metaphylactic: The use of mass medication to eliminate or minimize an expected outbreak of a disease; (iv) Growth promoters: Administered to animals to improve the growth rate and the food conversion. Table 1 reports the most commonly used antibiotics in aquaculture worldwide [15,16], in most European countries [17], and in the Mediterranean area [18]. 

## 3. Antibiotic Residues as Emerging Pollutants

Antibiotics are biologically active molecules that can exert toxic effects in the aquatic environment. These pharmaceuticals are considered contaminants of increasing concern, based on their common presence in different environmental contexts, and the lack of specific regulations for monitoring. The European Commission has established a Watch List of substances, including antibiotics, to induce the Member States to collect data on the concentration levels of antibiotics in the environment and to assess whether these substrates can be considered priority substances to be monitored. The antibiotics fluoroquinolone, ciprofloxacin, amoxicillin, azithromycin, clarithromycin, and erythromycin are included in the aforementioned Watch List [13]. Antibiotics are usually administered to aquaculture in the addition to feed or by immersion through closed containers [19]. After the addition of antibiotics, their concentrations increase in the water column, in sediments under the cages, and in fish. A portion of food modified with antibiotics and not ingested by the fish is deposited in the sediments below and near the aquaculture sites [20,21,22,23]. A percentage of 80% of the ingested antibiotics reach the environment with feces, in non-absorbed form. When antibiotics are absorbed, they are then excreted in urine and other secretions [21,24]. These antibiotic aliquots accumulate in sediments under and around aquaculture facilities and from there can then be carried by currents to distant sediment sites [25]. The half-life of antibiotics varies in sediments depending on the chemical structure [14,26,27]. Other variables influence the time of activity maintenance by antibiotics in sediments. These include sediments granulometry, content in organic matter, kind and intensity of water currents, levels of temperature, light intensity, pH values, and the activity of native microbial communities [14,28,29]. Traces of biologically active oxytetracycline can remain in sediments for long periods [26,28,30]. The antibiotics oxolinic acid and flumequine may persist close to fish farms for several months [28,31]. Concentrations of sulphonamides and trimethoprim are detectable and active in sediments for months [26,28,32]. The antibiotic florfenicol cannot be detected in the sediments a few days after its introduction, whereas the by-product, florfenicol amine, remains detectable for several months [28]. Tetracyclines can exert antibacterial activity even after adsorption to sediments, and remain reactive even in the presence of inhibitory cations such as Mg^2+^ and Ca^2+^ [33,34]). The antibiotics quinolones, sulfonamides, and tetracyclines can be absorbed by organic matter and can thus be accumulated in the environment. Antibiotics absorbed by sediments can be subject to a depletion of the activity of their principles [35]. In the Eastern Mediterranean Sea, investigations at Greek fish farms on the antibiotics oxytetracycline, florfenicol, and flumequine in the sediments beneath revealed that the only detectable antibiotic was flumequine at relatively low concentrations [36]. 

## 4. Induction of Antibiotic Resistance 

Antibiotic residues can select for resistant aquatic bacteria, promoting the spread of antibiotic resistance, even when concentrations were below the minimum inhibitory concentration (MIC) of bacterial strains of the community [37,38,39]. High frequencies of antibiotic-resistant bacteria have been reported in sites near aquaculture where antibiotics have been used, demonstrating that modified antibiotics in an aquaculture facility have a high potential to exert selective pressure and increase the frequency of antibiotic resistance in other environmental bacteria [40,41]. In the aquatic environment, 90% of aquatic bacteria show resistance to at least one antibiotic, and approximately 20% were multi antibiotic-resistant. In the case of simultaneous application of different antibiotics in aquaculture, multiresistant bacteria can develop. Bacteria carrying genes coding for novel antibiotic resistance mechanisms were moreover present [42]. Antibiotic resistance allows bacteria the survive high concentrations of antibiotics, conferring a selective advantage to members of communities carrying resistances. Resistant bacterial strains prevail over susceptible ones. An important and at the same time worrying aspect is that the antibiotics used in aquaculture include those used in human therapies, thus inducing resistance to these antibiotics [43]. 

In Mediterranean aquaculture facilities, misuse of antibiotics caused an increase in levels of antibiotics in the surrounding sediments and water column [18]. The use of antibiotics in the Mediterranean farmed European seabass (*D. labrax*) and gilthead seabream (*S. aurata*), describing oxytetracycline, oxolinic acid, flumequine, and potentiated sulphonamides evidencing methaphylaxis, a control treatment of a group of animals after the diagnosis of infection and/or clinical disease in part of the group, as the best practice for their use [44]. 

## 5. Transfer of Antibiotic Resistance between Bacteria

Aquaculture sites represent ‘hotspots for antibiotic-resistant genes’ [9,45,46,47]. Bacteria harboring different genes of antibiotic resistance can grow according to environmental features, spreading genes in different sites [48]. The aquatic environment may also contain human and animal bacterial pathogens, which act as agents in sharing genetic determinants between aquatic and terrestrial bacteria [23]. The set of mobile genetic elements in a genome is called a mobilome and can spread among aquatic bacteria. The mobilome comprehends naked DNA, insertion sequences, insertion sequence elements with common regions, integrons mobilized by plasmids, transposons, and integrative and conjugative elements, genomic islands, transposons and conjugative transposons, conjugative and mobilizable plasmids and bacteriophages, including phage-like elements designated gene transfer agents [23]. 

Horizontal gene transfer between aquatic and human pathogens is an important phenomenon involving antibiotic resistance genes. New genetic elements can thus enter into terrestrial bacterial communities, including human pathogens, the latter becoming more difficult to treat [49]. The same aquatic environments with aquaculture facilities can present unique conditions allowing horizontal gene transfer. One case is represented by the biofilms of aquatic bacteria attached to organic particulate matter, sediment clays and sands, and fish farm structures, combined with the large concentrations of bacteriophages and gene transfer agents in seawater, also allowing the transfer of horizontal gene and dissemination of antibiotic resistance [50]. In aquatic environments, horizontal gene transfer can be mediated by DNA generated by lysis of bacteriophage and by plasmids. Both naked DNA from bacteriophages and plasmids can contain antimicrobial resistance genes that may be expressed after entering bacteria. Aquatic bacteria such as *Vibrio* spp. resulted naturally competent for DNA uptake, allowing transformation to occur in the aquatic environment [23]. Antibiotic-resistant genes are characterized by a different persistence in the environment, depending on the plasmid or chromosomal origin. Higher mobility and higher concentrations characterize antibiotic-resistant genes originated from plasmid with respect to those from chromosomes [51]. 

Genes of antibiotic resistance are present as both intracellular and extracellular fractions of DNA in the environment. Intracellular and extracellular antibiotic resistance genes observe the same pathway in sediments in an aquaculture site and nearby sites, revealing the presence of connections between these different sites [52,53]. Characteristics of DNA molecules also impact the persistence of antibiotic-resistant genes in the extracellular environment [51]. Some extracellular antibiotic-resistant genes are more recalcitrant to DNA degrading enzymes (DNases) than others, likely due to their sequence and structural features [54]. Due to the higher surface charges and higher molecular flexibility, chromosomal DNA is more adsorptive than plasmid DNA [55]. Therefore, in the environment, extracellular chromosomal DNA can persist longer than extracellular plasmid DNA. In sediments, a lower detection frequency of extracellular DNA located on the plasmid is expected with respect to chromosomal DNA [56]. 

### 5.1. Conjugation

**Figure 1:**
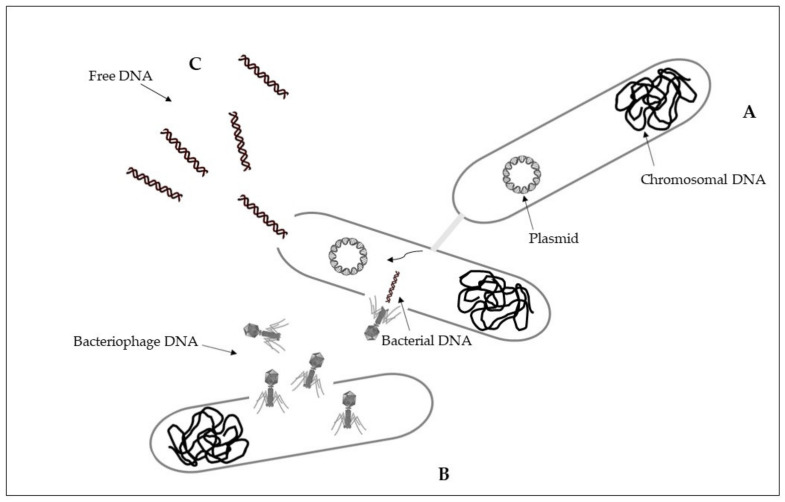
Horizontal gene transfer: (**A**), conjugation; (**B**), transduction; (**C**), transformation. See text for explanations.

Intracellular antibiotic-resistant genes may be disseminated via conjugation from cell-to-cell contact, and transduction because of infection of bacterial phages [57]. Conjugation is a horizontal gene transfer mechanism via cell to cell contact through junction due to pili or adhesins, by using a pore [58]. Conjugation is associated with plasmids that can transfer faster than a whole chromosome [59,60]. A percentage higher than 50% of known plasmids can be transferred by using conjugation as a horizontal gene transfer mechanism [61]. The process of conjugation may be present between the same bacterial species, but may also occur between unrelated populations characterized by a taxonomic distance, while at lower frequencies [58,59]. Plasmids and transposons can facilitate the conjugative transfer of antibiotic resistance by collecting antibiotic-resistant genes and carrying them to recipient cells [58,62]. Conjugation of antibiotic-resistant genes has been frequently reported in various environments, including soil, sediments, water, food, plant, animal, and clinical bacteria [59,61,63]. Conjugative transfer of multi-drug resistance among and across bacteria in different environments was showed [56,59] (Figure 1A). 

### 5.2. Transduction

Transduction is a horizontal gene transfer mechanism with bacteriophages that act as mediators for intracellular DNA transfer from an infected bacterial cell to a recipient bacterial cell [59,64]. Bacteriophages, or phages, are viruses that infect bacteria and are able to collect and transfer genes to a host bacterial [59,65]. Bacteriophages can transfer both chromosomal DNA and plasmid DNA [64,66]. Once DNA is transferred, it must be incorporated into the recipient chromosome by homologous recombination [64]. Some bacteriophages have a wide range of bacterial hosts and can move across different species [67]. The transfer of antibiotic resistance genes via phages for different bacterial species has been extensively described [68,69,70]. During the first phase, bacteriophages attach to the bacterial host and then inject their genome that has the capability to sequester the molecular machinery of the host bacterial cell to synthesize new phages. New phages can then lyse the host cell and spread to the environment [71] (Figure 1B).

### 5.3. Transformation 

Differently from intracellular DNA, extracellular antibiotic-resistant genes can enter the competent cells of non-resistant bacteria through the mechanism of natural transformation [57]. Extracellular DNA can originate from the lysis of dead cells and the secretion from live cells, representing a dynamic gene pool for natural transformation. Adsorption on sediment colloids, sand particles, clay minerals, and humic substances can protect extracellular DNA against enzyme nuclease attacks [72,73]. Natural transformation represents a direct uptake and integration of extracellular DNA [74]. It is essential that bacterial cells must first develop a regulated physiological state, a defined state of competence, for carrying out natural transformation [58,75]. Specific environmental conditions can stimulate competence development [59,76]. On the basis of the absorption of extracellular DNA by competent cells, there may be reasons such as nutrition, chromosomal DNA repair, and diversification of the genetic material for evolution [75]. Natural transformation requests the persistence of DNA in the extracellular environment, and the ability to resist degradation in environmental conditions. Higher efficiency of natural transformation is evident in the presence of longer extracellular DNA fragments with respect to the results obtained with smaller extracellular DNA fragments [77,78]. Once in the recipient cell, the new DNA must be integrated into the recipient bacterial genome in the case of chromosomal DNA, and be integrated or recircularized into a self-replicating plasmid, in the case of plasmid DNA [75]. Natural transformation through chromosomal DNA is thus more efficient than in the presence of plasmid DNA [56,58] (Figure 1C).

## 6. Transfer of Antibiotic Resistance from Aquaculture Bacteria to Human Pathogens

### 6.1. Quinolones

Antibiotics of the quinolones class are widely used in aquaculture, with traces found in aquaculture effluents, the water column, and sediments near aquaculture facilities. The quinolone concentrations found in these compartments are high enough to exert selective pressure on aquatic bacterial species. The latter can mix with species of different origins, favoring gene exchange and spreading resistance to antibiotics [79]. Quinolones are antibiotics with a broad spectrum against both Gram-positive and Gram-negative bacteria, including mycobacteria and anaerobes. They exert their actions by inhibiting bacterial nucleic acid synthesis through disrupting the enzymes topoisomerase IV and DNA gyrase, and by causing breakage of bacterial chromosomes. Mechanisms of resistance to quinolones provide mutations in the bacteria genes, as the mutation in genes encoding the DNA gyrase and topoisomerase IV targets, or other genomic alterations which alter topoisomerase targets, modify quinolone, or reduce drug accumulation by decreasing drug absorption or increasing drug outflow. Resistance to quinolones may result from the uptake of the plasmid gene from the environment or from other resistant strains [80]. Genes of quinolone resistance included in plasmid-mediated quinolone resistance (PMQR) are the followings: six *qnr* genes (*qnrA*, *qnrB*, *qnrC*, *qnrD*, *qnrS*, and *qnrVC*) encoding gyrase-protection repetitive peptides; *oqxAB*, *qepA*, and *qaqBIII* encoding efflux pumps; and *aac(6′)-Ib-cr* encoding an aminoglycoside and quinolone inactivating acetyl-transferase. Moreover, these genes can synergize with chromosomal *gyrA* and *parA* mutations conferring quinolone resistance [81]. The water-borne bacterial species *Shewanella algae* and *Vibrio splendidus* comprehend bacterial strains carrying *qnrA* and *qnrS* genes, respectively, and gene *qnrS* were detected in another water-borne strain, *Aeromonas* sp., the role of aquatic environments in the diffusion of such resistance determinants has acquired more and more importance [82]. Strains of both the aquatic bacterial species *Aeromonas punctata* subsp. *punctata* and *A. media* evidenced the presence of the *qnrS2* gene. The *qnrS2* gene was located on IncU-type plasmids in both isolates. When these plasmids were transferred into bacteria of the species *Escherichia coli*, they became highly resistant to quinolones and fluoroquinolones. The identification of plasmid-mediated *qnr* genes outside *Enterobacteriaceae* evidence a possible diffusion of those resistance determinants within Gram-negative bacteria [82]. The genes *qnrA*, *qnrB,* and *qnrS* for resistance to quinolones were found in the chromosome of marine bacteria isolated from an aquaculture facility in the Región de Los Lagos, Chile, and the same genes were detected in human pathogenic bacteria. The *qnrA* gene was, in fact, also found in the chromosome of two uropathogenic clinical strains of *E. coli* resistant to quinolones isolated from patients in a coastal area, bordering the same aquaculture region. The *qnrB* and *qnrS* genes were located in plasmids in two other *E. coli* strains isolated from the same clinical context [83]. Further investigations by sequencing *qnrA1*, *qnrB1,* and *qnrS1* genes in quinolone-resistant *E. coli* and in marine bacteria, both from Chile, were identical. A horizontal gene transfer between antibiotic-resistant marine bacteria and human pathogens was confirmed [56]. Concerning genetic elements of marine bacteria and uropathogenic *E. coli*, both evidenced class 1 integron with similar co-linear structures, identical gene cassettes, and similarities in their flanking regions. Investigations in a *Marinobacter* sp. marine isolate and in an *E. coli* clinical isolated strain, highlight sequences immediately upstream of the *qnrS* gene evidencing homology to comparable sequences of numerous plasmid-located *qnrS* genes. These investigations confirm that horizontal gene transfer between bacteria in diverse ecological locations is facilitated [83]. PMQR can be transferred horizontally among distantly related lineages and might play a role in maintaining resistance levels in bacterial populations in the presence of sub-inhibitory concentrations of antibiotics [84,85]. The same plasmid has been shown to play an important role for the spread of resistance genes not only quinolones but also for other antibiotics such as β-lactams and aminoglycosides. In fact, *qnr* genes are frequently carried along with β-lactamase determinants on the same plasmids. Moreover, it was evidenced that the prevalent *qnrA*, *qnrB*, *qnrS,* and *aac(6′)-Ib-cr* genes among quinolone and cephalosporin-resistant clinical isolates of *Klebsiella pneumoniae*, are in the association between PMQR genes with resistance to quinolones, cephalosporins, and aminoglycosides [86]. Aquaculture is a possible source of *aac(6′)-Ib-cr* and *qnrB2* in aquatic environments and *Enterobacteriaceae* were important hosts of these two genes. The ubiquitous bacteria, *Aeromonas* spp., served as vectors for *qnrS2* by means of IncQ-type plasmids. A water-human transmission by and via *Aeromonas* species was evidenced [87], and a *qnrS*-containing plasmid was identified in an *Aeromonas* sp. clinical isolate [88]. Before, genes *qnr* have only been reported in *Enterobacteriaceae* [89,90], with the one exception of a *qnrS*-containing plasmid found in environmental *A. punctata* subsp. *punctata* and *A. media* isolates [82]. A plasmid containing the *qnrS* was detected in an *Aeromonas* sp. clinically isolated strain for the first time [88], evidencing that a *qnrS2* gene was identified in a clinical isolate that was not within the *Enterobacteriaceae* family. The versatility of these determinants to spread among the different bacterial species with the consequent potential risk for human health became strongly evident [88]. From this evidence, the need to control the antibiotic resistance supervision of both clinical and environmental *Aeromonas* isolates has emerged [91,92]. Plasmid-encoded quinolone resistance genes (*qnrA, qnrB, qnrS,* and *aac [6′]-1b-cr*) were found in *E. coli* and in *Klebsiella* [93]. Genes for quinolone resistance were detected in the aquatic genera *Vibrio, Shewanella,* and *Aeromonas* and then those genes were detected in human and animal pathogens [23]. Bacterial strains belonging to the water-borne bacterial species *S. algae* and *V. splendidus*, evidenced the presence of *qnrA* and *qnrS* genes, respectively. The gene *qnrS* was identified in another water-borne strain *Aeromonas* sp., increasing evidence of the role of aquatic environments in spreading those resistance determinants [82]. Gram-negative bacterial species of the aquatic environment may be the reservoir of plasmid-mediated Qnr-like determinants, that seem closely related to the species *V. splendidus* [94]. The World Health Organization (WHO) designated *E. coli* that are resistant to fluoroquinolones as one of the nine pathogens of international concern [95]. A description of strains of *E. coli* in countries in the Mediterranean area is reported in Table 2. Values of percentages of *E. coli* resistance to fluoroquinolones originated mostly from hospitals, nevertheless, their origin with aquatic bacteria could be a real problem for both human and animal health, and a concern for the environment.

### 6.2. Tetracyclines

Tetracyclines are antibiotics inhibiting bacterial protein synthesis by preventing the association of aminoacyl tRNA with the bacterial ribosome [97]. Bacteria could use three strategies to become resistant to tetracycline: limiting the access of tetracycline to the ribosomes, altering the ribosome to prevent effective binding of tetracycline, and producing tetracycline-inactivating enzymes [98]. Tetracyclines are commonly used in human and veterinary treatment, with oxytetracycline that is permitted to be mixed with feed for fish [99]. In Japan, bacterial strains from aquaculture fish and bacteria from a close clinical facility exhibited a high similarity for genes of tetracycline resistance, suggesting that they may have originated from the same source. Laboratory experiments in which tetracycline resistance from marine strains of genera *Photobacterium, Vibrio, Alteromonas*, and *Pseudomonas* were transferred to *E. coli* by conjugation, confirmed the possibility of a transfer of resistance determinants from marine bacteria to bacteria inhabiting the human gut. Moreover, the same resistance gene profile in aquatic bacteria and in human clinical isolates was evidenced. The antibiotic-resistant genes for tetracycline resistance identified in fish pathogenic bacteria are common to those identified in human pathogens, and that bacteria from different environments, such as aquatic and clinical, can share the same antibiotic-resistant genes [21,99,100]. Tetracycline resistance determinants revealed in *Salmonella* spp. strains were detected in fish pathogens of the species *Vibrio anguillarum* [101,102,103]. Moreover, the DNA sequence of these antibiotic resistance determinants has an important DNA sequence similarity to a plasmid of *Pasteurella piscicida*, which is also a fish pathogen [102,103]. The independently evolved tetracycline-resistance determinant *tetG* was first discovered in aquatic bacteria [102]. In the genome of the animal pathogen *Chlamydia suis*, the *tetC* gene probably may have originated from the genome of the aquatic bacterium *Aeromonas salmonicida*, a pathogen of salmon [104]. The independently evolved tetracycline-resistance determinant *tetG* was first discovered in aquatic bacteria [102]. Tetracycline resistance genes were identified in marine sediments from resistance genes in bacterial plasmids from marine sediments that shared high identity with transposons or plasmids from human pathogens, indicating that the sediment bacteria can spread resistance genes from pathogens [17,84]. The same resistance gene profile has been described in both fish bacteria and human clinical isolates. About half of the antibiotic resistance genes identified in fish pathogens are common to those identified in human pathogens [99,100].

### 6.3. β-Lactams, Macrolides, Fosfomycin, Chloramphenicol, Colistin, Florfenicol

Commercial fish and seafood may act as a reservoir for multiresistant bacteria, facilitating the dissemination of antibiotic resistance genes. Broad-spectrum β-lactamase resistance genes, including *blaTEM-52, blaSHV-12*, as well as *cmlA, tetA, aadA, sul1, sul2*, and *sul3* were recovered in the faecal matter from *S. aurata* (Gilthead seabream) [105]. The β-lactam antibiotics inhibit the last step in peptidoglycan synthesis by acylating the transpeptidase involved in cross-linking peptides to form peptidoglycan. The targets for the actions of β-lactam antibiotics are known as penicillin-binding proteins. This binding, in turn, interrupts the terminal transpeptidation process and induces loss of viability and lysis, also through autolytic processes within the bacterial cell [106]. Although bacterial resistance to β-lactams mostly expresses through the production of β-lactamases, other mechanisms are involved. Following are the mechanisms of resistance: (i) inactivation by the production of β-lactamases; (ii) decreased penetration to the target site as resistance in *Pseudomonas aeruginosa*; (iii) alteration of target site penicillin-binding proteins as penicillin resistance in pneumococci; (iv) efflux from the periplasmic space through specific pumping mechanisms [107]. Other examples of resistances with aquatic origin include the gene of fosfomycin resistance isolated from the aquatic environment, the widely disseminated emerging *floR* gene of human pathogens, and the chloramphenicol resistance genes *catII*, *catB9* and *catB2*, originating respectively from aquatic bacteria of the genera *Photobacterium*, *Vibrio*, and *Shewanella* [23]. Additionally, the plasmid-associated colistin resistance mediated by the *mcr-1* gene appears to be another transmissible antibiotic resistance determinant that might have originated in the aquaculture facilities [17,108,109]. The macrolide resistance genes *mef(C)* and *mef(G)* in *Vibrio* spp. and *Photobacterium* spp. strains appear to have an aquatic origin [110]. Indeed, resistance genes have been found on transferable plasmids and integrons in pathogenic bacterial species of the genera *Aeromonas*, *Yersinia*, *Photobacterium*, *Edwardsiella*, and *Vibrio*. Class 1 integrons and IncA/C plasmids have been widely identified in important fish pathogens (*Aeromonas* spp., *Yersinia* spp., *Photobacterium* spp., *Edwardsiella* spp., and *Vibrio* spp.) and are thought to play a major role in the transmission of antimicrobial resistance determinants in the aquatic environment. The identification of plasmids in terrestrial pathogens (*Salmonella enterica* serotypes, *E. coli*, and others) which have considerable homology to plasmid backbone DNA from aquatic pathogens suggests that the plasmid profiles of fish pathogens are extremely plastic and mobile and constitute a considerable reservoir for antimicrobial resistance genes for pathogens in diverse environments [111]. The florfenicol determinant, *floR*, was detected for the first time in the fish pathogen *Vibrio damsela* [112]. This molecular evidence strongly suggests that there was a horizontal transmission of antibiotic resistance determinants from bacteria in the aquaculture environment to a human and terrestrial veterinary pathogen [102,103]. The epidemiology of the dissemination of *S. typhimurium* DT104 also suggests this pathogen could have been spread by fish meal as has happened with the *Salmonella* Agona that originated in Peru several years ago [102,113]. This process illustrates the potential role of the transport of antibiotic-resistant bacteria as an alternative mechanism responsible for the spread of antibiotic resistance determinants from the aquatic environment to the terrestrial environment [103,114]. As another example, *V. cholerae* of the Latin American epidemic of cholera that started in 1992, appeared to have acquired antibiotic resistance as a result of coming into contact with antibiotic-resistant bacteria selected through the heavy use of antibiotics in the Ecuadorian shrimp industry [102,115]. Multiresistant bacteria were evidenced in aquaculture contexts, including potential human pathogens strains of the genera *Vibrio*, *Pseudomonas*, and *Salmonella*, evidencing the possibility to transfer genetic determinants of resistance to pathogenic bacteria, both in water and in sediments [116]. Pathogens bacterial strains in aquaculture, belonging to the genera *Aeromonas*, *Edwardsiella*, *Flavobacterium*, *Lactococcus*, *Photobacterium*, *Pseudomonas*, *Renibacterium*, *Streptococcus*, *Vibrio*, *Yersinia*, were pointed out [117].

## 7. Antibiotic Resistance in the Mediterranean Basin

In the Mediterranean area, fish farms of European seabass (*D. labrax*) and Mediterranean gilthead seabream (*S. aurata*) are mostly present, and antibiotics were added by feed amendments [44]. In Western Mediterranean coastal sediments from the vicinity of Ligurian Sea coastal fish facilities, Gram-negative bacterial strains have been found showing high resistance to the antibiotics ampicillin and streptomycin. Multiple resistances were found in the same strains. Antibiotic resistance patterns close to fish farming showed high incidences of quinolone, tetracycline, and penicillin-resistant bacterial populations [118]. In the Mediterranean area, antibiotic-resistant bacterial pathogens isolated from sediments near a fish farm in Greece, were compared with bacterial isolates in Italy and France, including *Vibrio* spp., *Pseudomonas* spp., *Aeromonas* spp., and *Pasteurella piscicida*. Greek, Italian and French aquaculture sites allowed isolation of bacterial strains evidencing similar antibiotic sensitivity pathways, with resistance to erythromycin, kanamycin, and streptomycin, and sensitivity to most of the other antibiotics tested. Resistance was also demonstrated for some isolates towards the potentiated sulphonamides [119]. Bacterial strains *Vibrio* spp., *Pseudomonas* spp., and *Photobacterium damselae* ssp. *piscicida* isolated from gilthead seabream (*S. aurata*) from an aquaculture facility in southwestern Spain, in the Mediterranean region, evidenced high levels of antibiotic resistance [120]. Bacterial strains of *V. anguillarum* isolated from Greek fish farms evidenced multiple antibiotic resistance [121]. Multiple antibiotic resistance pathways have been highlighted in native marine bacterial strains isolated from fish farms located along the Adriatic Sea (Italy) and identified as *Vibrio* spp. Resistance to tetracycline (17%), trimethoprim-sulfadiazine (7%), and trimethoprim (2%) resulted in the most frequently obtained patterns [122,123]. Pathogenic halophilic strains *Vibrio parahaemolyticus* and *V. vulnificus* evidencing resistance to lincomycin, were moreover isolated from seafood in Italy [124]. High frequencies of *Aeromonas* spp. contamination in *S. aurata* from the Italian coast was pointed out, with elevated biodiversity among isolated bacterial strains. The bacterial strains showed high resistance to sulfadiazine, amoxicillin, ampicillin, erythromycin, cephalotin, streptomycin, and trimethoprim antibiotics. It was evidenced that almost all *Aeromonas* spp. strains showed multiple antibiotic resistance and potentially pathogenic species for humans were included, evidencing the capability to transfer via the food chain the genes responsible for antibiotic resistance to human pathogens [125]. More than one hundred bacterial strains were isolated from samples of *S. aurata* in an aquaculture site in Portugal. Included in the isolates, *Enterobacter* spp. and *Pseudomonas* spp. strains resulted in resistance to ertapenem and meropenem, antibiotics used in serious clinical infections. Several antibiotics for which resistance was found in these isolates appear in the World Health Organization list of “critically important antimicrobials” and “highly important antimicrobials” for human medicine [126]. In Mediterranean aquaculture, and in particular the Greek fish farm facilities, several registered antibiotics are currently available against bacterial infections, including tetracyclines, quinolones, and fluoroquinolones, potential sulfa, penicillin, and chloramphenicol derivatives. Oxytetracycline and quinolone drugs, with oxolinic acid and flumequine, are the most widely used in Mediterranean aquaculture [18]. A comparison was made of the distribution of tetracycline resistance genes in neighboring Greek fish farms and coastal environments. The *tetA* and *tetK* genes were detected in both habitats, while the *tetC* and *tetE* genes were present in fish farms and in wastewater, and *tetM* was found in fish farms and coastal sites. Some isolates were obtained, highlighting the presence of resistance genes *teth*, *tetC*, *tetK,* and *tetM* in the main part. The isolates were assigned to the genera *Stenotrophomonas*, *Acinetobacter*, *Pseudomonas*, *Bacillus,* and *Staphylococcus*. Isolated strains showed the presence of IncP plasmids, harboring tetracycline resistance genes (*i.e., teth*, *tetC*, *tetE,* and *tetK*), and the dissemination of IncP plasmids was evidenced. Based on these results, it has been shown that tetracyclines resistance genes from seawater sites have spread in bacterial communities, via broad-spectrum host plasmids [127]. The onset of antibiotic resistance in the environment and in non-clinical environments, highlight the importance of the investigation of antibiotic resistance in environmental contexts [128]. The use of antibiotics in aquaculture can lead to the emergence of antibiotic resistance in bacteria that are pathogenic to humans, posing a serious threat to public health [129]. In 2015, antibiotic-resistant bacteria increased in the European area, causing over 33,000 human deaths. Furthermore, this number is projected to increase by 2030, due to rapid socioeconomic growth and population expansion [130,131]. The emergence of antibiotic resistance in bacteria poses a serious threat, with a reduction in the use of antibiotics in aquaculture feed that is absolutely required by 2030 [132]. In Table 3, a description of antibiotic-resistant bacteria, and eventual genes for antibiotic resistance, in aquaculture sites of different countries of the Mediterranean basin was reported. The presence of antibiotic-resistant bacteria from aquaculture facilities in the Mediterranean area, highlighting serious concerns for human and animal health and for the environment, is evidenced.

## 8. Antibiotic Resistance and Temperature

Antibiotics act on specific targets of bacterial cells, by altering the cellular balance. Similar changes are evident when evaluating the effects of temperature on bacterial cells. Interestingly, modifications in bacterial cells caused by antibiotics coincide with those caused by temperature increases. Due to the commonality of actions of antibiotics and temperature, a further increase in antibiotic resistance may be experienced [154]. Aminoglycosides are a class of antibiotics damaging the cell structures both by binding to the ribosomes and by determining an increase of misfolded proteins in cells and qualitatively similar changes may be caused by temperature increases and by the stresses of heat shock [155,156]. The aminoglycosides gentamicin and tobramycin induce heat shock genes in *Bacillus subtilis* and *P. aeruginosa*, respectively [157,158]. The aminoglycoside antibiotic kanamycin can induce a similar response, with misfolded proteins, in *E. coli* [159], when the induction of several heat shock proteins, including GroEL, GroES, and DnaK in bacterial cells of the species *E. coli* was observed [160]. The aminoglycosides antibiotics kanamycin and streptomycin induce the protein-expression profile in *E. coli*, similar to that induced under heat shock [160]. A similarity between the effects of temperature-induced protein unfolding and aminoglycoside-induced misfolded proteins due to translational misreading was highlighted [159]. In bacteria of the *E. coli* species, a similar trend was shown between the cellular effects of exposure at 46 °C and the effects deriving from exposure to the aminoglycoside antibiotics gentamicin, tobramycin, and streptomycin [161]. A commonality of effect between antibiotic and temperature was also highlighted in the case of the aminoglycoside antibiotic streptomycin, which increased expression of heat shock proteins DnaK (Hsp70) and GroEL (Hsp60) in the opportunistic human pathogen *Acinetobacter baumannii* [162]. Further investigations showed that *A. baumannii* cells pretreated for 30 min at 45 °C grew better when subsequently exposed to streptomycin, compared to cells pretreated at 37 °C [163,164]. Resistance to aminoglycoside streptomycin in *A. baumannii* is due to chaperonins acting against aminoglycoside-induced protein misfolding [165]. In *E. coli* it has been evidenced that antibiotics nitrofurantoin and trimethoprim damage DNA with effects similar to heat, at a temperature of 44 °C. In heat stress, DNA repair mechanisms have also been involved. In *E. coli*, investigations of thermotolerant proteins discovered that various proteins codified by genes *dnaQ*, *holC*, *priA*, *ruvA*, and *ruvC*, involved in DNA repair are needed for growth at 47 °C [166,167].

### 8.1. Effect Cross-Protection

Mutations conferring resistance to temperature stress can thus induce resistance to antibiotics and vice versa, a phenomenon known as collateral resistance or cross-protection [168]. The effects of collateral resistance or cross-protection follow the principle that bacterial communities have a genotypic highly phenotypically plastic during adaptation in changing environments [169]. The term cross-protection refers to acquired resistance to specific stress after previous contact with another stressor agent. This pathway has been highlighted for various stress combinations and it has been observed in many species across the tree of life, including microorganisms [170]. Cells of *E. coli* were exposed to high-temperature for 2000 generations in an experiment of cross-protection [168]. A portion of 10% of the experimental lines showed de novo mutations after hundreds of generations, acquiring resistance to rifampicin, although the same antibiotic never came into contact with bacterial cells during the experiment. In this case, the selective pressure was represented by a high temperature of 42 °C. The mechanism of cross-resistance emerged because both temperature and rifampicin are active on the same target of selection, the RNA polymerase, deputed to transcription of DNA into RNA and to gene expression control. Mutations in the active site of the RNA polymerase led to changes in gene expression that were adaptive under high-temperature stress [171]. Resistance to rifampicin depends on a collateral effect of heat-stress adaptation, as temperature increases cause amino acid substitutions that alter the active site of the RNA polymerase, preventing the binding of rifampicin to the same deputed site. Resistance to the antibiotics trimethoprim and nitrofurantoin in some high-temperature adapted strains was moreover evidenced [161]. Additional mutations contributing to the adaptation to thermal stress favor resistance to the antibiotic rifamycin, identified by *rpoB* gene mutations [167]. The temperature effects can have a role in the evolution of antibiotic resistance, as they evidenced long-term heritable effects conferred by genetic modifications. Antibiotic resistance can originate due to spontaneous mutations or can be acquired as a consequence of horizontal gene transfer. It was supposed that temperature has effects on horizontal gene transfer, with the latter that is more common at 30 °C than at 25 °C [172]. 

### 8.2. Modifications Induced by Temperature

Temperature influence in antibiotic resistance, and related changes, are then maintained in the microbial community. As an example of structural modification, the increased temperature may favor biofilm formation, as in a marine bacterium strain of the genus *Roseovarius* growing at 33 °C instead of the optimal temperature of 25 °C, giving rise to a biofilm. The reason for biofilm formation resides in an adaptation to high-temperature, with an increase of biofilm formation at the air–liquid interface. Temperature increases thus modify physiological features that select for different adaptations, as in the case of biofilms. These adaptations may result in a higher tolerance to antibiotics, with broader implications for marine bacteria that are experiencing warmer seawater temperatures caused by climate change [173]. The temperature allows for antibiotic resistance mutations to appear early on. In fact, the temperature can potentiate adaptive evolution by increasing the phenotypic and genotypic variation in a population, according to the concept of evolvability [174]. To make the evolution of adaptive mutations more likely, it is necessary to increase the rate of spontaneous mutations. In this context, the thermal shock response and the general stress response lead to an increase in mutagenesis [175]. Once a mutation of resistance arises, it can proceed towards fixation in the community or be eliminated. The progress of a mutation depends on how efficient and beneficial it is in a given environment. Temperature, by increasing mutagenesis, can favor the adaptation of a mutation and its evolution [176]. In the presence of antibiotics, resistance mutations can save the life of bacterial cells. However, when the pressure of antibiotics is no longer present, resistance mutations can become a cost to the cell, resulting in less efficiency of the cell functions. In the absence of antibiotics, the temperature can lower these costs while maintaining antibiotic resistance, based on a mechanism similar to compensatory mutations [177,178]. Antibiotic resistance mutations are not necessarily costly or deleterious under conditions of thermal stress with no antibiotic present [168,179]. Mutations in the RNA polymerase beta subunit (*rpoB*) that confer resistance to rifampicin in *E. coli* are beneficial at high temperatures of 40 °C [179] and 42 °C [168]. The *rpoB* mutations for rifampicin resistance confer advantages in restoring gene expression patterns from a burden state toward an unburned one, thus restoring the physiology present at equilibrium [171]. In cells of *E. coli*, high temperatures increase the chance and magnitude of positive selection, thus explaining the geographic patterns in evolutionary rates and understanding contemporary evolution under global warming [180]. In the tree of life, based on analyses of ribosomal RNA sequences, hyperthermophiles are placed near the common ancestor, in the presence of high temperatures. The temperature must thus constitute one of the oldest adaptations in nature [181]. Furthermore, the thermal shock response, which is a cellular mechanism that deals with the effects of high temperatures, is present in all domains of life and is highly conserved [182]. The first antibiotics appeared between 2 billion and 40 million years ago and probable changes in bacterial cells in response to heat stress were then co-opted to cope with antibiotic stress. Antibiotic resistance is therefore a natural phenomenon that arose before the modern selective pressure of the clinical use of antibiotics [183]. The fact that adaptations to changes in environmental temperature evolved before responses to antibiotics were confirmed, as the same mechanisms that confer resistance to temperature increases act on antibiotic resistance [184,185]. To respond to stress, a cell makes an important investment in terms of genetic information, protein synthesis, time to evolve the process, and energy. It is therefore not advantageous for a cell to develop a unique response for each individual stressor, but it is more beneficial if it can co-opt similar pathways to respond to different stressors [161,169,186]. When a bacterial cell undergoes high temperatures, after the thermal shock that causes a transient effect in which the thermal stress proteins are encoded, there is a period of phenotypic acclimatization [187,188]. In the event that environmental stress persists, the bacterial cells undergo an accumulation of mutations so as to induce the entire microbial community towards a real adaptation. This process occurs thanks to genetic changes that give rise to new characteristics or new physiological functions, for example, resistance to antibiotics [171,189,190]. Evolution can represent novelties with unknown aspects that can open ecological opportunities and favor biodiversity [189]. In *E. coli* cells, the evolution of a new trait involves genetic information or environmental conditions, rendering it compatible with evolution. A mutation with subsequent opportunities for further refinement mutations is therefore required, which can benefit from an emerging trait so that a newly colonized niche can be fully utilized by bacteria of the *E. coli* species [190].

### 8.3. Epigenetic Modifications

Epigenetics plays a role in the onset of antibiotic resistance among bacteria [191]. Epigenetic inheritance mediated antibiotic resistance in bacteria may be a mechanism that can drive evolution [192]. The epigenome is represented by a set of molecular modifications that overlap the genome and drive gene expression. Epigenetic modifications have a character of reversibility and can create adaptive, phenotypic plasticity of gene expression under environmental stress, and may increase the potential to adapt to environmental change [193], or buffer organisms against deleterious environmental effects [194]. A mechanism of epigenetic modification is DNA methylation, a process by which methyl groups are removed from S-adenyl methionine and are usually covalently added to cytosine, 5′ to guanosine, within a dinucleotide CpG site [195,196]. A role of epigenetic mechanisms was evidenced in bacterial and archaeal thermal stress tolerance [197,198], with critical implications for microbial processes in a world facing climate change [199]. Recent investigations suggest a pivotal role for epigenetics in the development of antibiotic resistance in bacteria. The methylation of adenines and cytosines can influence mutation rates in bacterial genomes, thus modulating antibiotic susceptibility. Epigenetic mechanisms evidence the emerging roles in antibiotic resistance [191]. Some studies are starting to indicate a role of epigenetic with critical implications for microbial processes in a warming world [199].

## 9. Climate Change and Antibiotic Resistance

Climate change influences antibiotic resistance onset through temperature increases. A study about relationships between temperature and antibiotic resistance was conducted in the United States, collecting data of temperature from 1980 to 2010 and collecting data from hundreds of hospitals and laboratories across 41 states concerning percentages of antibiotic-resistant bacterial strains. The attention focused on resistance in three widespread pathogens of concern, the Gram-negative *E. coli* and *Klebsiella pneumoniae* and Gram-positive *Staphylococcus aureus*. This study highlighted that for every 10 °C increase in minimum temperature, there was a significant increase in the percentage of resistant strains, corresponding to 4.2% in *E. coli*, 2.2% in *K. pneumoniae*, and 2.7% *S. aureus* [200]. This was the first study that highlights and demonstrates that the temperature increase predicted by climate change will cause an increase in antibiotic resistance in pathogenic bacteria [200]. Another study, conducted in Europe from 2000 to 2016, evaluated the effects of temperature on antibiotic resistance in 28 European countries. Bacterial pathogens were tested in the presence of four classes of antibiotics, evidencing a long-term effect of ambient minimum temperature on rates of increase of antibiotic resistance. Across all antibiotic classes for the pathogens, *E. coli* and *K. pneumoniae*, in European countries with 10 °C warmer ambient temperatures, more rapid increases in antibiotic resistance over the years of the investigation were pointed out. Antibiotic resistance increases were included in a range between 0.33%/year and 1.2%/year [154]. The trend of increases in temperature was the same as that of values of percentages of antibiotic-resistant bacteria, evidencing a correlation between warmer temperatures with an increase in the overall presence of resistant bacteria. The study also showed a correlation between warmer temperatures and increased rates of change for levels of the same resistant bacteria. A careful evaluation of the past two decades in terms of temperature values and antibiotic resistance allowed a temporal analysis of antibiotic resistance throughout most of Europe. The careful and meticulous measurements across time in this study over the last two decades allowed a temporal analysis of resistance throughout most of Europe. Importantly, the rapid rate of resistance was observed even after controlling for overall antibiotic consumption and human population density that affect resistance [200]. Objective 2 of the World Health Organization (WHO) global action plan on antimicrobial resistance cites as follows: “*Strengthen the knowledge and evidence base through surveillance and research*” [201]. Considering that the 2017 EARS-Net surveillance report highlights that, as in previous years, for the period of 2013–2016, “a north-to-south and a west-to-east gradient is evident in Europe,” with a general increase of antibiotic resistance prevalence over this gradient, particularly for the Gram-negative bacteria surveyed [202]. Following this information, a study was conducted that included seven countries distributed on the EARS-Net conceptual gradient represented by Portugal, Spain, Cyprus, Ireland, Germany, Norway, and Finland. This investigation pointed out that antibiotic use and environmental temperature have a role in spreading antibiotic resistance in the environment [203]. In fact, the antibiotic-resistant genes burden was significantly higher in southern countries (Portugal, Spain, and Cyprus) than in the northern countries (Germany, Norway, and Finland) [203]. Countries from the north and from the south of Europe differed in the rate of antibiotic use in humans, but also in other, probably non-negligible factors, such as temperature, precipitation, or antibiotic use in pets and in livestock. The latter could give residues that may contribute to an overall increase of antibiotic or antibiotic resistance load in that region [203]. In the context of aquaculture, it is important to consider the sustainability of aquaculture with respect to environmental changes. Investigations of how global warming and antimicrobial resistance affect aquaculture show that the antibiotic resistance indices of aquaculture are correlated with those of human clinical bacteria, temperature, and climate vulnerability of countries. Interestingly, infected fish have higher mortality at warmer temperatures. In this context, developing countries are likely to have much higher risks, with a major impact on human health, in addition to that on the aquaculture sector, highlighting the need for urgent action. Therefore, sustainable solutions are needed to minimize the use of antibiotics, enabling system resilience [131]. 

Changes in temperature can lead to the development of antibiotic resistance in bacteria which, while not all pathogenic, represent a reservoir of antibiotic-resistant genes that can be passed on to human pathogenic bacteria. It is therefore essential to study the emergence and persistence of antibiotic resistance in both clinically relevant pathogens and non-pathogenic environmental bacteria. To this end, a multidisciplinary approach is needed that evaluates the effects of temperature as a whole [167]. Relationships between climate change, including global warming, and the occurrence of antibiotic resistance in bacteria of aquaculture was evidenced by studying temperature effect on the mortality of aquatic animals infected with pathogenic bacteria spread in aquaculture contexts. It was observed that fish contaminated by pathogenic bacteria pointed out higher mortality events at warmer temperatures [167]. The Multi-Antibiotic Bacterial Resistance Index (MAR) showed that most countries have elevated MAR indices of aquaculture-related bacteria, which in turn are correlated with MAR indices obtained from human clinical bacteria, with human health problems [167].

## 10. Climate Change in the Mediterranean Area

The Mediterranean Sea is a semi-closed regional sea, mostly deep, which has different areas given the complex morphology of the Mediterranean region from which it originates [204]. The Mediterranean region is located in a transition zone between the arid climate of North Africa and the temperate and rainy climate of Central Europe and is affected by the interactions between mid-latitudes and tropical processes. Based on these characteristics, even relatively minor changes in general circulation can lead to substantial changes in the Mediterranean climate. The Mediterranean is therefore a region potentially vulnerable to climate change induced by variables such as the increase in greenhouse gas concentrations [205,206]. The Mediterranean region has been subject in the past to major climate change and is considered as an important “Hot-Spot” concerning climate change [207,208]. The Mediterranean climate is mild and humid in winter, hot and dry in summer. In winter it is mainly affected by Atlantic storms moving west and converging on the coasts of Western Europe. The trend of the Mediterranean winter climate, and in particular that of precipitation, depends on the North Atlantic Oscillation (NAO) on its western areas and on the eastern Atlantic (EA) [209,210]. Furthermore, the Southern El Niño Oscillation (ENSO) significantly affects the variability of winter precipitation in the eastern Mediterranean, continuing to exert its influence on spring and autumn rains in the Iberian Peninsula and northwestern Africa [211]. In addition to storms that originate from the Atlantic, these atmospheric phenomena may have an internal origin in the Mediterranean storm region, in correspondence with cyclogenetic areas such as the lee of the Alps, the Gulf of Lyon, and the Gulf of Genoa [212]. During the summer, high pressure and descending motions dominate over the Mediterranean region, with dry conditions in particular over the south. Mediterranean climate variability of summer is connected with monsoons from Asia and Africa [211]. 

The climate of the Mediterranean, in addition to processes on a planetary scale and teleconnections, is influenced by local processes induced by the complex physiography of the area and by the presence of a large body of water (the Mediterranean Sea). In this context, the Alpine chain is a strong factor in modifying synoptic and mesoscale travel systems and the Mediterranean Sea is an important source of moisture and energy for storms [205,212]. The regional climate signal at small spatial scales is regulated by the complex topography, coastline, and vegetation cover of the Mediterranean region [205]. In addition, anthropogenic and natural aerosols of central European, African, and Asian origin can reach the Mediterranean, possibly influencing its climate characteristics [211]. Following the interactions of processes at a wide range of spatial and temporal scales, the climate of the Mediterranean is characterized by a great diversity of features, resulting in a variety of climate types [205]. Climate change caused by greenhouse gas, evidenced a northward shift of the North Atlantic winter storm with an intensification of the North Atlantic Oscillation [206]. As a consequence, it is possible to observe an increase in precipitation in northern Europe and reductions of precipitations over many parts of the Mediterranean. A strong decrease of precipitation over the whole Mediterranean basin was evidenced, in particular over the western Iberian Peninsula, southern Turkey, the Near East, and Egypt, with differences of more than 30% [206]. In the Mediterranean area, climate change will have serious effects on extensive aquaculture, shellfish culture, intensive inshore aquaculture, and aquaculture in coastal lagoons [131]. The Mediterranean will show a depletion of precipitation, reaching a probable loss of precipitation locally which will reach 40%. This will strongly reduce water resources that will constrain the ability of the region to develop and grow food, affecting millions of already water-stressed people and threatening the stability of this area. Wintertime Mediterranean circulation trends can be seen as the combined response to two independent forcings: robust changes in large-scale, upper-tropospheric flow and the reduction in the land-sea temperature gradient that is characteristic of this region [213]. In Southern Europe, changes can lead to a temperature increase of 4–5 °C with longer drought periods, resulting in increasing desertification, and a decrease in crop yields. In areas of Western and Atlantic Europe, changes of 2.5–3.5 °C with drier and hotter summers are expected. In Central Europe, an increase of 3–4 °C, higher rainfall, and floods are envisaged. Northern Europe would expect a mean temperature increase of 3–4.5 °C, with a significant increase in precipitation of 30–40% [214]. Based on climate change projections over the Mediterranean region, a whole view presents a situation with a substantial drying and warming of the Mediterranean region, especially in the warm season with precipitation decreases around −25–30% and warming increases of 4–5 °C. An exception to this behavior is represented by an increase of precipitation during the winter time in the region of Alps, in the northern Mediterranean basin [215]. The Mediterranean is included among the most responsive regions to global climate change. It must consider that the Mediterranean is a transition area between the temperate climate of central Europe and the arid climate of northern Africa, such changes have the potential to profoundly modify the climate characteristics of the Mediterranean [215]. An increase in global atmospheric temperature of 2 °C will likely be accompanied by a reduction in summer rainfall of around 10–15% in southern France, northwestern Spain, and the Balkans and up to 30% in Turkey and Portugal [216]. Scenarios with 2–4 °C temperature increases in the 2080s for Southern Europe would imply widespread decreases in precipitation of up to 30% (especially in spring and summer months) and a switch to a lack of a frost season in the Balkans [217]. According to the 5th report from the Intergovernmental Panel for Climate Change (IPCC) [218], the prevalence of summer peak temperatures, heatwaves, and periods dominated by high water temperature is expected to increase markedly in the Mediterranean region in the coming years [219]. 

In the Mediterranean Basin, human society and the natural environment have co-evolved over several millennia, experiencing significant climatic variations and preparing the environment for diverse and culturally rich communities. The region is characterized by a complex morphology of mountain chains and strong land-sea contrasts, a dense and growing human population, and various environmental pressures. Observed rates of climate change in the Mediterranean Basin exceed global trends for most variables. Basin-wide, annual mean temperatures are now 1.4 °C above late-nineteenth-century levels, particularly during the summer months [220]. For each of the most recent decades, the surface of the Mediterranean Sea has warmed by around 0.4 °C [221]. Considering the ‘Paris-compliant’, a probable increase in global warming of 1.5 °C, with a maximum of 2.2 °C at a regional level, is expected [222]. Aquaculture facilities and interactions with human safety in the Mediterranean must be seriously considered. This geographical area has individualities in both the hydrological and physicochemical characteristics and the forms of aquaculture activities [10].

### Mediterranean-Type Ecosystem 

Description of the characteristics of the Mediterranean area can be extended to a group of Mediterranean-type ecosystems representative in the world. Mediterranean-type ecosystems have distinctive climatic regimes and characteristic mild, wet winters and hot, dry summers, which occur in five regions of the world: California; Central Chile; the Mediterranean basin; the Cape region of South Africa; and in the southwest and south of Australia. The five Mediterranean climate regions of the world host important and significant levels of plant diversity and endemism, allowing to design all five regions as “biodiversity hotspots”. These regions with a Mediterranean climate are also internationally recognized as some of the most threatened ecosystems in the world. The five Mediterranean regions are estimated to have important changes in biodiversity by 2100 due to their susceptibility to climate change [223].

## 11. One Health and Antibiotic Resistance in Aquaculture

The concerns of antibiotic resistance due to fish treatment with antibiotics in aquaculture have an impact on human health. This is an example of communication among different comparts and sheds light on the actions carried out in one sector can have repercussions, even heavily, in other contexts. It is therefore clear that in order to address these issues, an overview is required, it is required to act globally and to take into consideration the various facets of the problem. Transmission of antibiotic resistance to bacteria of the environment, then passing to human pathogens, represents serious concerns. Areas utilized by aquaculture activities and, thus, the Mediterranean area are threatened by a serious problem, further worsened and magnified by climate change. Antibiotics misuse and climate change are the causes at the base of these phenomena. In the presence of such difficulties, an approach aimed to consider the health of fish, human beings, and the aquatic ecosystem, and encouragement of environmentally friendly measures of disease prevention could give basic support to solve the problem. It is necessary to adopt measures reinforcing efforts dealing with antibiotic resistance by developing new therapeutic agents if headway is to be made against the increasing problem of antibiotic resistance in human and veterinary medicine [104]. This kind of attitude is defined as a One Health approach: *One Health is a collaborative, multisector, and transdisciplinary approach—working at the local, regional, national, and global levels—with the goal of achieving optimal health outcomes recognizing the interconnection between people, animals, plants, and their shared environment*. One Health describes a principle recognizing that human and animal health are interconnected, that diseases are transmitted from humans to animals and vice versa and must therefore be tackled together. This also includes the environment, representing another link between humans and animals and at the same time a potential source of new bacterial resistance to antibiotics [224]. The One Health approach must be adopted for a strategic sector such as aquaculture to cope with that challenge in the respect of the sustainable development goals of Agenda 2030. The strategies regarding antibiotic resistance in aquaculture are based on guidelines for the judicious use of antibiotics in veterinary contexts and on the principle that similar strategies used for reducing antibiotic use in terrestrial farm animals should also be used in aquaculture [225]. The One Health action plan focuses on combatting increasing antibiotic resistance by reducing infections caused by resistant bacteria [201,226]. 

### Good Practices to Be Adopted

Reduction of antibiotic use in aquaculture include implementation of good aquaculture practices that provides the appropriate environmental conditions, by improving hygiene and preventive measures at the fish farm level; avoid overuse of antibiotics in human and veterinary medicine; increase the research of new antibiotics or alternatives; avoid transmission of resistant bacteria from animals to humans either directly or through the food chain; increase awareness and knowledge on antibiotic resistance in professionals. Moreover, appropriate feeding, antibiotics sensitivity testing before treatment, development of specific disease surveillance programs to prevent possible outbreaks, implementing hygiene by cleaning and disinfecting units between production cycles, keeping separate equipment, boots, and clothes for each unit. Biosecurity measures must be moreover observed. Vaccination against some etiological agents of infectious diseases resulted as effective for reducing antibiotic use and consequently antibiotic resistance [227]. The One Health approach, with the involvement of all sectors and aspects of antibiotic resistance, including human medicine, veterinary medicine, animal husbandry, agriculture, research, environment, and trade, could give important results [228]. The new action plan was developed based on the 2011 One Health document and was launched in 2017. Its main goal is to preserve the possibility of effective treatment of infections in humans and animals by providing a framework for continued, more extensive action to reduce the emergence and spread of antibiotic resistance and to increase the development and availability of new effective antimicrobial agents inside and outside Europe [229].

Considering that climate change, via temperature increases, affects antibiotic resistance onset, there is the need to respect intentions of reducing causes of climate change. Thus, correct and diminish climate change parameters as temperature, is needed. Among the purposes of One Health, high importance is given to the environment. The protection of the environment, as well as that of other contexts, can only be achieved by adopting the One Health method which provides for cooperation between the various disciplines [230]. Assessing the effects of climate change following principles of One Health, an integrated view of the health of humans, animals, and plants could reduce the effects of climate change [230,231,232,233]. The 2015 *Lancet* Commission concluded that the response to climate change could be “the greatest global health opportunity of the 21st century” [234]. The *Lancet* Countdown aims to track the health impacts of climate hazards; health resilience and adaptation; health co-benefits of climate change mitigation; economics and finance; and political and broader engagement [234]. In the case of climate change, an integrated One Health approach can unify scientific disciplines, policy-making, and local knowledge by engaging non-academic stakeholders and different academic disciplines to act together locally, nationally, and globally to address and solve health problems related to climate change [235].

**Figure 2:**
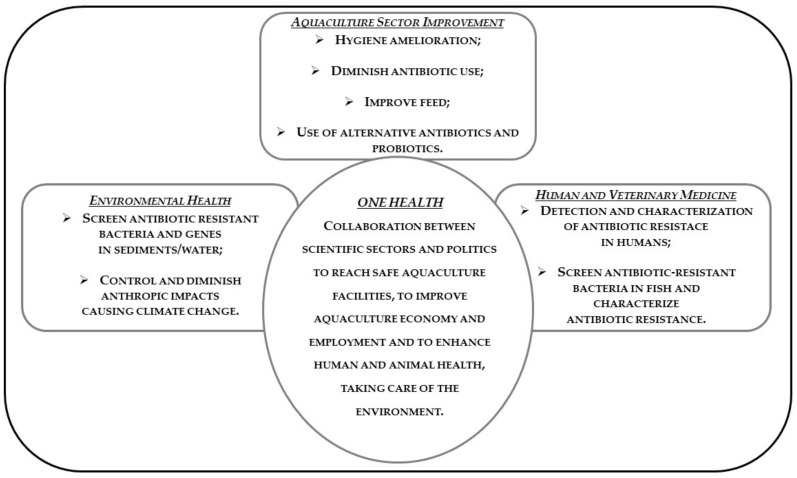
One Health possible approach for the antibiotic resistance concern in the Mediterranean area.

One Health approaches show clear advantages over conventional public and animal health approaches both for adaptation to and mitigation of climate change, and for surveillance of antibiotic resistance [236]. A problem can be represented by the lack of collaboration between the different sectors [237]. Existing legal systems are adequate to regulate professional services in all sectors, but mechanisms to work better together and to communicate fully between the involved sectors of human health, animal health and environmental health are of basic importance [238]. Resolution of complex global health problems requires interdisciplinary, expertise and cooperation from governmental, nongovernmental, and educational agencies. One Health refers to the collaboration of multiple disciplines and sectors working locally, nationally, and globally to attain optimal health for people, animals, and the environment. One Health offers the opportunity to share interests, set common goals, and drive toward teamwork, with the aim to benefit the overall health of a nation. This approach drives innovations that are important to solve both acute and chronic health problems and offers a synergy of the different systems, obtaining improved communications, evidencing based solutions, development of a new generation of systems-thinkers, improved surveillance, decreased lag time in response, and improved health and economy [239]. Due to the variability of the Mediterranean basin, interventions of different disciplines, involving local authorities and inhabitants, could give important support in a One Health approach. Figure 2 reports a scheme of the possible approaches that can be adopted in view of a One Health intervention about the concern of antibiotic resistance in the Mediterranean basin.

## 12. Conclusions

In the face of undoubted advantages in economic and social terms, aquaculture in the Mediterranean basin involves some critical issues, one of which is represented by resistance to antibiotics. A link between antibiotics utilization in animals, the emergence of resistant bacterial strains, and the transfer of resistance to human pathogens have been highlighted. Furthermore, climatic changes and temperature increases lead to an adaptation of the bacteria which causes a significant onset of antibiotic resistance. The Mediterranean and its characteristics are a readable representation on a larger scale, also considering that the characteristics of the Mediterranean area can be extended to a group of representative Mediterranean-type ecosystems in the world. These regions with a Mediterranean climate are also internationally recognized as some of the most threatened ecosystems in the world due to their sensitivity to climate change. Considering the recommendations of the 2030 Agenda based on Global Development Goals, it is necessary to follow the principles of sustainability for a useful and beneficial growth of the resources provided by aquaculture, respecting human and animal health and in harmony with the environment. To achieve these objectives, it is necessary to reduce the use of antibiotics in aquaculture and to face the reduced support of antibiotics by adopting the most strict hygienic rules and clean fish farms, introduce new antibiotics against infections, and develop eventual vaccines to reduce the probability of infections in fish. In the meantime, it is necessary to drastically diminish the causes of climate change and to monitor and treat the problem in a global approach, meaning considering the problem from the environmental, medical, and social points of view. The One Health approach, with the involvement of medical, veterinary, and environmental aspects, can offer higher possibilities to solve these problems. The area of the Mediterranean Sea presents very particular local realities, due to differences among countries bordering this area. It is, therefore, important that both the intervention to reduce the use of antibiotics and to counter the effects of the increase in temperature and climate change involve local authorities and residents, as they have a deep and consolidated knowledge of regional realities. After the local approach, regional, national, and global levels can be reached according to the principles of One Health.

New insights and new research directions could include the use of new antibiotics, for instance from marine microorganisms, to be applied in aquaculture, avoiding the utilization of the same antibiotics deputed to human therapies. As for recommendations, it is important to decrease the use of antibiotics in aquaculture, improving the care of fish farms, and promoting the hygiene of the facilities. Actions to control climate change need to be considered, including depleting CO_2_ production as the main temperature control intervention, avoiding the dramatic 3–4 °C increase by 2030. These two actions can together improve the final conditions of the Mediterranean area in terms of public health.

## Figures and Tables

**Table 1:** The most commonly used antibiotics in aquaculture.

Class	Antibiotic
Worldwide [15,16]	
aminoglycosides	streptomycin
amphenicols	florfenicol
β-lactams	amoxicillin, ampicillin
diaminopyrimidines	ormethoprim
macrolides	erythromycin
quinolones	oxolinic acid, flumequine, sarafloxacin, enrofloxacin
sulfonamides	sulfadimethoxine
tetracyclines	tetracycline, oxytetracycline
Most European Countries [17]	
amphenicols	florfenicol
macrolides	erythromycin
quinolones	sarafloxacin
sulphonamides	boosted with trimethoprim or ormethoprim
tetracyclines	oxytetracycline
In Mediterranean Aquaculture [18]	
quinolones	oxolinic acid, flumequine
tetracyclines	oxytetracycline

**Table 2:** *Escherichia coli* isolated strains resistant to fluoroquinolones in countries of the Mediterranean area.

Country	Mean Percentage (%)	Range Percentage (%)	Year	Isolated Strains	Source
Bosnia and Herzegovina	29	23–35	2016	215	CAESAR
Croatia	29	26–32	2017	1150	EARS-Net
France	17	16–18	2017	13,328	EARS-Net
Greece	34	32–36	2017	1464	EARS-Net
Italy	47	46–48	2017	6945	EARS-Net
Lebanon	45	33–57	2016	65	GLASS
Portugal	30	29–31	2017	6424	EARS-Net
Spain	33	32–34	2017	5557	EARS-Net
Tunisia	19	13–29	2017	78	GLASS
Turkey	55	53–57	2016	3670	CAESAR

European Antimicrobial Resistance Surveilance Network (EARS-Net); Central Asian and Eastern European Surveillance of Antimicrobial Resistance (CAESAR); Global Antimicrobial Resistance Surveillance System (GLASS) [96].

**Table 3:** Bacteria isolated from aquaculture in the Mediterranean area, antibiotic resistance and antibiotic-resistance genes.

Country/Area	Isolated Bacteria Species/Genus/Family/Order/Class	Antibiotic-Resistance	Antibiotic-Resistant Genes	References
Algeria	*Vibrio alginolyticus, V. cholerae, V. fluvialis, V. hollisae*			[133]
Croatia	*Aeromonas* spp.			[134]
Eastern Adriatic	*Vibrio* spp.	flumequinone, chloramphenicol, oxytetracycline		[135]
Egypt	*Aeromonas* spp.	chloramphenicol, kanamycin, azithromycin		[136]
Egypt	*Pseudomonas anguilliseptica*			[137]
Egypt	*Aeromonas hydrophila*	penicillin, erythromycin		[138]
Egypt	*Enterobacteriaceae*	cephalosporins, carbapenem	*blaKPC (blaCTX-M-15, blaSHV, blaTEM, blaPER-1)*	[139]
France	*Flavobacterium psychrophilum*			[140]
France	*Yersinia ruckeri*			[141]
Greece	*Acinetobacter* spp., *Bacillus* spp., *Pseudomonas* spp., *Staphylococcus* spp., *Stenotrophomonas* spp.	tetracycline	*tetA, tetK, tetC, tetE, tetM*	[127]
Italy	Enterococci	ampicillin, gentamicin	*tetM, tetL, tetO, ermB, mef*	[142]
Italy	*Aeromonas* spp., *Photobacterium* spp., *Shewanella* spp., *Vibrio* spp.	tetracycline, flumequine, trimethoprim		[122]
Italy	*Photobacterium damselae* ssp. *piscicida, Vibrio fluvialis, V. alginolyticus, V. parahaemolyticus, V. metschnikovii*	ampicillin, carbenicillin, kanamycin, cefalothin		[143]
Italy	*Aeromonas* spp.	ampicillin, amoxicillin, cephalothin, erythromycin, streptomycin, sulfadiazine, trimethoprim		[125]
Italy	*Shewanella algae*, *Vibrio* spp.	beta-lactams, quinolones, tetracyclines, macrolides, polymyxins, chloramphenicol, fosfomycin, erythromycin	*blaOXA-55-like,* *blaAmpC, mexB-OprM, mdtG, mdlB,tet34, tet35, tetR, eptA, cat, mdtL*	[46]
Lebanon	*Streptococcus pneumoniae*	polymyxins, chloramphenicol, fosfomycin, erythromycin		[144]
Spain	*Aeromonas* spp., *Salmonella* spp., *Vibrio mimicus, V. furnissii.*	oxytetracycline, nitrofurantoin, oxacillin, sulfomethoxazole/trimethoprim		[145]
Spain	*Flavobacterium psychrophilum*	oxytetracycline, florfenicol		[146]
Spain	*Aeromonas salmonicida*	nalidixic acid, oxytetracycline		[147]
Tunisia	*Vibrio alginolyticus*	ampicillin, erythromycin, kanamycin, cefataxime, streptomycin, trimetoprim		[148]
Tunisia	*Escherichia coli*	tertracycline, streptomycin, ampicillin, trimethoprim, sulfamethoxazole	*tetA-tetB*	[149]
Turkey	*E. coli*, coliforms, fish pathogens	sulfamethoxazole, ampicillin, sulfamethoxazole, imipenem, aztreonam	*ampC, blaCTX-M1, tetA, sul2, blaTEM*	[150]
Turkey	*Y. ruckeri*		*floR, sulI, tetC, tetD, tetE*	[151]
Turkey	*Y. ruckeri*	erythromycin, florfenicol, sulfonamide, tetracycline, trimetophrin	*ermB, ermY, floR, su/I, suffll, tetA-tetG*	[152]
Turkey	*Aeromonas media, A. rivipollensis, A. salmonicida, Bacillus pumilus, B. zhangzhouensis, Hafnia alvei, Kluyvera intermedia, Pantoea* spp., *Pseudomonas* spp., *P. protegens, Staphylococcus* spp., Gammaproteobacteria, Betaproteobacteria, Enterobacteriales, Burkholderiales	sulfamethacin, sulfamerazine, erythromycin, tetracycline		[153]

## Data Availability

The Center for Disease, Dynamics Economics and Policy. Resistance Map: Antibiotic Resistance. 2021. Available online: https://resistancemap.cddep.org/AntibioticResistance.php (accessed on 11 February 2021).
